# Young adults understanding and readiness to engage with palliative care: extending the reach of palliative care through a public health approach: a qualitative study

**DOI:** 10.1186/s12904-021-00808-0

**Published:** 2021-07-28

**Authors:** Anita Mallon, Felicity Hasson, Karen Casson, Paul Slater, Sonja McIlfatrick

**Affiliations:** Institute of Nursing and Health Research, School of Nursing, Ulster University, Shore Road, Newtownabbey, BT37 0QB Northern Ireland

**Keywords:** Palliative Care, Public Health, Young Adult, Attitudes, Qualitative

## Abstract

**Background:**

Moving palliative care from a solely clinical focus to a more population based and community orientated approach is the hallmark of a much advocated public health approach to palliative care. Young adults are a vital cohort of the public, yet their understanding of palliative care has not been investigated. This study aimed to explore young adults’ understanding of palliative care and identify factors that influence their engagement.

**Methods:**

A purposive sample of young adults (n = 24) aged 18–29 years were recruited from one UK University. Semi-structured interviews were undertaken face to face or via telephone or Skype between November 2017 and February 2018. Thematic analysis using a framework approach and underpinned by a socioecological perspective was used to analyse the interviews.

**Results:**

Three thematic categories were identified relating to intrapersonal and interpersonal influences, cultural and social influences and organisational and public policy influences. Palliative care was understood as supportive comfort care, delivered in the absence of cure, associated with the end of life and specifically focused on death and dying. Negative attitudes related to the context of care, which represented a static and hopeless situation. Whilst some reported positive attitudes, potential engagement was seen to be governed by a lack of knowledge and protective cultural norms. In terms of demonstrating readiness to engage with palliative care, participants requested clear information and suggested a normalising of palliative care through the education system.

**Conclusion:**

Young adults in this study were ready to find out more about palliative care and identified social media as a platform upon which to engage this population. However, their perception of a society that views palliative care as a subject for those directly affected, creates a barrier to engagement. This study identified the ingredients of a public health message and mediums for disseminating the message. However, findings also suggest that a cultural shift is required to recognise the potential of engaging young adults in health issues that cross the life span, empowering them not only as individuals but as vital members of community and society.

**Supplementary Information:**

The online version contains supplementary material available at 10.1186/s12904-021-00808-0.

## Introduction

Despite global recognition of palliative care under a human right to health [1] and as an essential component of primary care that should be available to all, [2] there remains a significant imbalance between palliative care need and access to services [3]. A reorientation of palliative care towards more community based and population based models of care has been advocated with the aim of extending the reach of palliative care [4]. It is suggested that public health systems can help frame palliative care and provide both structures and resources that will enable its integration into all health and social care systems and into civil society [5]. However, the success of a public health approach to palliative care is based on two assumptions: (a) that the public has sufficient knowledge to accept palliative care as a priority health issue that is relevant to their present lives, and (b) that the public have sufficient understanding to accept a role in how palliative care develops and responds to this unprecedented need. In a study looking at the early impact of the World Health Association (WHA) resolution on the strengthening of palliative care, varied public understanding of palliative care, societal taboos and inhibitions were identified as factors that inhibited the integration of palliative care [6]. Empirical studies suggest that the public have poor knowledge of palliative care that is focused on care in terminal illness, with little change in understanding over time [7]. The need to target specific groups of the public to determine their understanding of palliative care and to explore unexplained differences within demographic groups has been advocated [8, 9].

It can be argued that young adults are a powerful cohort of the ‘public’ in their ability to facilitate behaviour change [10] and often reflect broader societal trends in attitudes [11]. However, young adults (18–30 years) have participated less in surveys relating to public awareness and perceptions of palliative care and demonstrated less knowledge of palliative care [12–14], with more reticence in using the term identified among younger researchers and academics in palliative care [15]. Studies looking specifically at young adults’ understanding of palliative care have focused almost exclusively on students in health care settings with a view to future caring roles. Multiple influences such as older age, [16] being female, [17, 18] having a personal belief system [19] and previous experience of palliative care [20–23] were reported to positively influence attitudes and perceptions of palliative care among students studying health care courses. Yet the gulf between need and access to palliative care presents a pressing societal issue that affects everyone. Only a small number of surveys have looked at knowledge and perceptions of palliative care with a broader population of young adults; these have focused on aspects of palliative care such as awareness and the affiliation of palliative care with cancer [24, 25]. A first phase of this study reported elsewhere [26] found that familiarity and experience with palliative care was the most significant predictor of young adults’ knowledge of palliative care. Less negative attitudes to palliative care have been reported with increased knowledge among a university student population [27]. There is a dearth of qualitative inquiry in studies to date and a lack of overt theoretical guidance to explain measured constructs, limiting insight into the context and experience that may help explain how public knowledge of palliative care has not developed in line with the discipline of palliative care. In this study we aimed to explore the understanding of palliative care held by young adults and identify what influences their understanding. Determinants may then be identified that will inform a public health approach aiming to promote greater public engagement with palliative care.

**Fig. 1:**
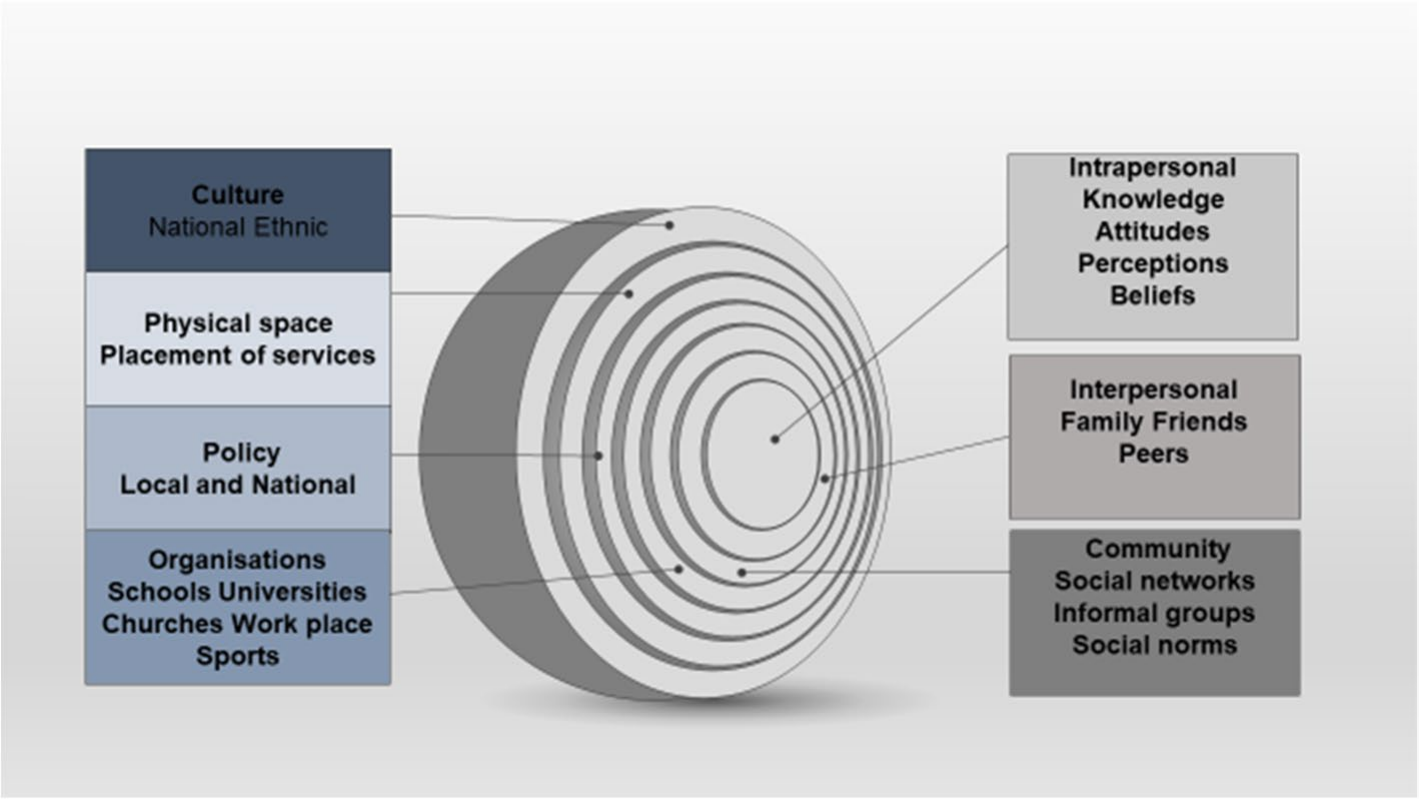
Schematic presentation of concentric layers of the Socio-Ecological Model (SEM) 28 depicting societal levels of influence on health and behaviour

A socioecological perspective underpinned the study and the Socio-Ecological Model (SEM) [28] provided a framework for exploring the multiple levels of influence on young adults’ understanding of palliative care. These include societal level influences at the intrapersonal, interpersonal, community, organisational, public policy, physical environment and cultural levels with each level impacting other levels providing targets for possible future public health interventions. Figure 1 illustrates the embedded systems framework of the Socio- Ecological Model [28].

### Study design

A qualitative design using semi-structured interviews was employed. The COnsolidated criteria for REporting Qualitative research (COREQ) [29] was used to guide the report.

### Setting, sample and recruitment

Participants who completed an online survey (n = 859) were invited to take part in this second phase of data collection. Participants had to have completed the survey which required them to be aged 18–29 years inclusive and to be registered at the University situated in a region of the United Kingdom. To enhance participation, a draw for travel vouchers was offered. In total 78 participants forwarded an expression of interest through a separate link embedded in the survey. They were then contacted with participant information and enquiries were made relating to time and mode of interview. Twenty four out of the 78 contacted agreed a time and place to participate in the interviews.

### Data collection

Interviews were undertaken between November 2017 and February 2018. These were undertaken by the first author (AM) and ranged between 20–50 min, with an average time of approximately 30 min. The interview guide was informed by a previous survey, [26] literature and the theoretical framework (see Additional file 1). The guide focused on understanding of palliative care, barriers and facilitators to engagement and the nature of messaging required to promote better understanding of palliative care for younger people. To enhance participation, participants were offered a choice of medium which included face-to- face (n = 17), Skype (n = 5) and telephone (n = 2). All interviews were audio taped, with field notes indexed to aid transferability of the data.

### Data analysis

All interviews were transcribed verbatim. Thematic analysis using a framework approach [30, 31], enabled both inductive and deductive labelling of themes. This hybrid approach meant that data labelling was not restricted by the theoretical constructs and an openness was maintained to the unexpected data in participant accounts [32] .Table 1 outlines how the 5 step Framework process [30] framed the analysis and the measures undertaken to ensure the trustworthiness of the interview data. The analysis was carried out by the principal investigator (AM) and independent analysis was undertaken by a second investigator (FH). Interpretation was discussed among all team members. Possible biases were mediated by a supervisory team with a wealth of experience in palliative care (SM, FH) and public health and research skills (KC, PS) who challenged interpretation. A reflexive journal was also kept by the researcher.

**Table 1:** Steps in thematic analysis using ‘Framework’ [30]

**Steps in Analysis**	**Procedures**
Familiarisation and transcription	Preliminary analysis of recordings, field and reflective notes allowed for the iterative adjustment of the interview schedule with the inclusion of further probes. This step enabled an overall view of the data and a list of 72 preliminary codes were initially devised
Identifying a framework	The framework categories were based around the interview schedule which was underpinned by the literature, theoretical model and informed by the survey data. The thematic matrix initially represented the general content of the interviews and provided the ‘thick description’ that is needed to interpret the data relating to other contexts [33].
Indexing	The matrix provided a structure enabling discussion and collaboration among a diverse research team. A challenge was the overlapping nature of the data. While data relating to feelings and emotions can cause problems relating to categorisation, [34] the theoretical perspective of the SEM had sufficient breadth to ensure the data were not restricted, initially the same data were coded in several categories
Charting	This step involved the summary and charting of the data within the categories allowing between-case and across-case analyses allowing for refinement of categories and themes
Mapping and interpretation	As a test of authenticity, the researchers read across the data in each case to check that they got a sense of the interview looking between themes for patterns. [31] First attempts at association and explanation were drafted by the researcher and discussed with the team. Following discussion and redrafting of the analysis, a consensus in interpretation was reached

### Ethical considerations

A study protocol was submitted to the University Institute of Nursing and Health Research Governance Filter Committee for ethical approval. Approval was granted on the 9th August 2017. Informed consent was given in writing prior to the interview and recorded for the Skype and telephone interviews. Each participant was given a numerical code and all identifiers were removed.

## Results

Findings are presented under three main categories aligned to the societal levels of the Socio- Ecological Model^; 28^ Intrapersonal and Interpersonal influences, Cultural and Social influences and Organisational and Policy influences.

### Participant characteristics

Twenty-four participants took part in the interviews. These included nine males and 15 females with a mean age of 23 years (range 18–28 years), comprised of both undergraduate (UG) (n = 16) and postgraduate (PG) (n = 8) students. Participants were from a range of faculties and courses; Faculty of Life and Health Sciences (n = 10); Faculty of Arts Humanities and Social Sciences (n = 7); Faculty of Computing, Engineering and the Built Environment (n = 4) and The Business School (n = 3). Participants were undertaking a range of graduate and undergraduate programmes including Drama, Animation, Tourism, Film and Theatre, Business, Social Work, Radiography, Oncology and Radiography, Biomedical Science, Construction, Computers, Engineering, Speech and Language Therapy, Biology and Environmental Science, no two participants were from the same programme. There was one non- Caucasian participant. Ten of the participants reported a religious affiliation. Table 2 presents additional participant information.

**Table 2:** Additional participant information

Participant code	Gender	Age group years	Undergraduate (UG)/ Postgraduate (PG)	Faculty of study
P01	M	22–28	PG	Life and Health Sciences
P02	F	18–21	UG	Business School
P03	F	18–21	UG	Life and Health Sciences
P04	F	22–28	UG	Arts, Humanities and Social Sciences
P05	M	22–28	PG	Life and Health Sciences
P06	M	22–28	UG	Computing, Engineering and the Built Environment
P07	M	22 -28	UG	Computer Engineering and the Built Environment
P08	F	22–28	UG	Life and Health Sciences
P09	M	22–28	UG	Arts, Humanities and Social Sciences
P10	F	18–21	UG	Arts, Humanities and Social Sciences
P11	F	22–28	PG	Arts, Humanities and Social Sciences
P12	M	18–21	UG	Arts, Humanities and Social Sciences
P13	F	22–28	PG	Business School
P14	F	18–21	PG	Business School
P15	M	22–28	UG	Computing, Engineering and the Built Environment
P16	F	18–21	UG	Life and Health Sciences
P17	M	18–21	UG	Life and Health Sciences
P18	F	18–21	UG	Life and Health Sciences
P19	F	18–21	UG	Arts, Humanities and Social Sciences
P20	F	18–21	UG	Life and Health Sciences
P21	F	22–28	PG	Life and Health Sciences
P22	F	18–21	UG	Life and Health Sciences
P23	F	22–28	PG	Arts, Humanities and Social Sciences
P24	M	22–28	PG	Computing, Engineering and the Built Environment

### Exposure and experience of palliative care

The majority of participants (n = 20) had heard of palliative care prior to participating in the survey. Most participants reported no experience of palliative care, with some unsure if care their relatives had received was palliative care. Personal experiences of palliative care related primarily to older relatives cared for in nursing homes and end of life care at home and in hospital. Palliative care formed part of courses, with three participants reporting experiences of palliative care delivery while on clinical placements. Two participants had heard about palliative care from friends and family in health care and one participant had discussed the issue with a family friend who had received palliative care in a specialist unit.

#### Intrapersonal and Interpersonal Influences

This theme relates to how knowledge, beliefs, individual characteristics and the influence of family and friends impact understanding of palliative care, including suggestions on how these characteristics could positively influence future initiatives aimed at promoting positive attitudes and engagement with palliative care.

There was no single reference point for how participants were exposed to and developed understanding of palliative care. Exposure was primarily through friends, family and study, ranging from a vague recollection of the term being mentioned to providing care at the end of life. Some retrospective understanding of palliative care was reported. Several participants suggested that, while they initially assumed they had no experience of palliative care, their older relatives *may* have received it, but they were unaware of it at the time. Exposure to palliative care through study and personal experience allowed participants to form a base upon which to understand palliative care. However, an undergraduate nursing student with experience of palliative care delivery in the community and a  postgraduate student in the Faculty of Computing, Engineering and the Built Environment with no comparable experience reported  similar understandings of palliative car; as professional care delivered to people at the end of life;

*‘Whenever they are not able to be independent…they’re maybe sleeping a lot of time. On things like syringe drivers or not getting out of bed’. (P20)*

*‘My impression would be that someone with palliative care would be bed bound and potentially unresponsive or close to unresponsive’. (P24) *

Across faculties the perception was that older people were generally the population to which palliative care would have most relevance and cancer was the most frequently related illness. Supportive comfort care was cited by all participants as a primary goal of palliative care;

*‘(Palliative care) provides comfort and safety, I suppose. Knowing that they’re not by themselves and they’ve got people there taking care of them and taking care of their needs’. (P14).*

Health professionals were viewed as the main palliative care providers, the ‘reasoning’ (P15) for care and the ‘capabilities’ (P18) of the family were deemed as important considerations in determining who delivered the care. Shortfalls in support networks were a reason for more professional input. Bereavement care as a core tenet of palliative care was given more consideration by those who reported experience of personal loss. Those who were less familiar with palliative care were more likely to see the supportive role of palliative care ending at death;

*‘(Palliative care is…) Just whenever the health professionals are looking after terminally ill patients. You know, whenever they get a diagnosis that they’re terminally ill. It would be the aftercare after that, up until they pass away…’* *(P13).*

It was interesting to note some differences in perceptions based on the gender of the participant. Those identifying with a male gender were more likely to report perceptions of palliative care as an inactive type of care representing an irredeemable and static situation. The belief that palliative care represented something that could not be fixed or helped was reported by several male participants who felt that interaction would be futile and that further discussion was not required;

*‘If there is a treatment in place, like we’re going for surgery, then it’s if the surgery works out, do one thing. If the surgery doesn’t work out, then there’s other options. Where perhaps with palliative care, there’s a sense of there’s only one option at the end of it and it is what it is and then there’s nothing to discuss in many ways’. (*P24*).*

Physical help to maintain comfort was seen to replace other aspirations held by people in good health, again relating to the association of palliative care with dependency;

‘*Your quality of life in palliative care is different to your quality of life to the everyday lay person. So it changes from wanting to travel to certain places to an everyday basic bodily functions’. (P05).*

Male participants were more likely to want to ‘manage’ the situation or relationships in the absence of being able to change or fix the situation and knowledge was key to this;

‘*Knowledge provides the know how to manage the relationship’. (P01).*

“*Get a mission plan so to speak as to how I am going to deal with what is round the corner’*. *(P15).*

Attempts to make palliative care relevant and engage young adults required more information that would empower them to make decisions relating to accessing palliative care. Making the beneficial outcomes of palliative care explicit was considered a way of engaging people in a type of risk–benefit analysis;

‘*If people actually understood more the personal outcomes (of palliative care) that when these steps are introduced what result does that actually have for the patient. Is that actually quite a positive time in the patient’s life, given the circumstances, compared to, if it wasn’t implemented, what the option is?’ (P24).*

Gender was important in making public messaging relevant. A participant described how the Lifeline campaign resonated with him;

‘*The Lifeline one is maybe more so directed towards people of my age group and especially men as well. If you think of the adverts and stuff, you know, you’ve the man on the radio and stuff, there’s a man on it on the TV and things, it’s all of directed – a lot of it is directed towards men*’. *(P09).*

At the interpersonal level participants expressed interest in enhancing their knowledge of palliative care. They suggested that knowledge would give them confidence, allow for meaningful engagement and positively influence their beliefs. While participants said they were comfortable talking about palliative care, it was more as an abstract issue or a hypothetical scenario;

‘*As a young person I would feel quite comfortable. At the same time, I never really had someone that was affected by it’. (P06).*

Young adults were perceived to be more open to discussion and developing opinions than older populations who may be;

*‘Set in their ways and it is very hard to try and change their opinion and if you try and change their mind, they put up a wall you can’t get over, my generation does not have that wall, maybe we don’t have that wall built up yet*’. *(P10).*

There was a belief by some who had experience of death and grief that hypothetical consideration of such an important subject could lessen its devastating impact;

***‘*** *It is one thing to talk about when you are in good health and it is fine and hypothetical but I think when you are in the midst of something it is a lot more daunting’. (P23)*

When asked what they wanted to know, participants suggested that a general overview of palliative care should be available with information tailored to those with specific needs. Information should relate to two areas: (1) Palliative care structure, services, locality and access and (2) How to manage relationships and have meaningful engagement with those requiring palliative care and their families;

*‘I suppose exactly what it is and how you can get in touch with people if you feel that someone needs it’. (P16)*

*‘The way that it is dealt with I guess, the different facets of the care and how to support someone going through without the disease, the issues hanging over the relationship’.(P12).*

For some participants their beliefs were informed by the experience of palliative care in clinical settings which was limited to the end of life. They identified a need for supplementary information as a resource to enable a broader perspective of the subject;

*‘I don’t know anything about the different types of palliative care. I have only seen it…, whenever it is catering for the patient’s end of life’. (P21)*

Highlighting how caring for ‘the palliative patient’ is an everyday occurrence for generalists, a nursing student related her experience as ‘*learning on the job*’ which needed to be supplemented with a firm knowledge base*;*

*‘I would like more information because that is something we are dealing with nearly every day…it would be good to have something to refer to, for your own understanding’. (P20)*

### Cultural and Social Influences

The inextricable link between palliative care and death and dying predominated the interviews. While palliative care itself was considered innocuous, a culture of fear and discomfort provided the fragile context within which palliative care was positioned. Attempts to resolve the discomfort meant that social norms that dictated when, how and where young adults engaged with the subject of palliative care were followed, despite some realisation that these norms limited possible attributes that young adults could bring to care giving and in some cases were detrimental to health.

Discomfort came from a perceived lack of skills which made participants fearful of embarking on emotional conversations and added to a sense of responsibility for causing upset. The idea that palliative care should form part of ordinary conversation was an anathema to some participants who did not see any immediate need or opportunity for discussion;

*‘No one wants to talk about it sitting in a café, over a coffee its’ like ‘So what are you going to do when you’re diagnosed with terminal cancer? What a way to kill the conversation’. (P07).*

Participants used terms such as ‘f*earful*’, ‘*terrifying*’ and ‘*distressing*’ when discussing death, dying and grief and its effect on the person and the family unit. Despite this, the social taboo with compliance to certain timeframes within which it was acceptable to discuss issues relating to palliative care was kept alive ‘*obviously time has to pass before you’d be able to maybe talk about it*’ (P20). The reluctance appeared to be avoidance of discomfort, intrusion and a lack of knowledge as to how to communicate with those who grieve for the loss of persons and loss of health.

The context of palliative care highlighted a societal awkwardness in communicating with people who were grieving. One participant reflected on the accepted and detrimental norm that allowed people to focus on the positive issues that are outside the person’s immediate grief experience. She felt this added to the stress experienced by her personal loss and put an extra burden on her to facilitate those positive conversations. Reflecting on a societal need to put a positive spin on what is a devastating situation, this participant emphasised that loss needed to be acknowledged;

‘*Like isn’t that fab that they got to be in the place that they wanted to be in when they passed away, give them (the family) the recognition that it is tough and hard and even though they maybe went the way they wanted to go that it is still really like shit’. (P23).*

A culture that separated young adults from the responsibility of conversation and decisions relating to palliative care was apparent. Participants perceived themselves to be ‘bystanders’ or actively excluded from conversations that related to care decisions, serious illness and death. Vague memories of conversations with ‘hints’ given perpetuated the idea that palliative care was not an issue for them as young people. It was ‘*over there … for old people’* (P22), not what young adults would have to think about. The lack of presence of palliative care in their everyday life and the young psyche that concentrates on what is immediately relevant and profitable meant that palliative care could wait;

*‘I’d rather be doing uni coursework, that (palliative care) doesn’t apply to me now. I have an exam on whatever and that is not going to help me now’. (P19)*

A protective norm was perpetuated where participants struggled with the idea of introducing palliative care to younger children. They could see the benefit of normalising the subject but did not want to spoil the children’s happiness;

*‘They don’t need to have concerns at that age. So it’s bringing in something that might bring them down’. (P02)*

Lack of engagement resulted in participants feeling ill prepared for navigating social situations relating to caring in severe illness and rituals associated with death;

*‘I think sometimes because people don’t know an awful lot about it, that in itself makes people shy away from it, because they don’t really know what to say, or they’re afraid of maybe saying the wrong thing’.(P09).*

Participants looked to older adults, often within families, to guide their behaviour. Describing attending a wake, a participant related how in the absence of knowledge his behaviour mimicked that of others;

*‘You just followed the person in front, you didn’t know what to do. By having more knowledge of this type of care, if I was to enter a house you would know how to speak and what way to conduct yourself, because you would understand what is actually happening in the other room*’. *(P06).*

The perception prevailed that palliative care would not be of interest or understood by those who had not been directly affected by illness, death or grief;

*‘I think particularly at this age…, that there’s not really that much discussion or knowledge of it and I think that’s totally understandable, because unless you have a family member, or unless you work in that field, like you’re not going to see it. It’s not going to cross your mind and you’re not going to consider those things really’. (P03).*

The inhibitive nature of social norms that dictate when, how and with whom discussions relating to palliative care could take place was a prominent theme in participant accounts. As a private matter, it was not openly discussed; conversations relating to palliative care were perceived as intimate between close family members and talked about in hushed tones in small groups with shared experiences. A participant suggested that, unlike hospice, palliative care ‘*is shoved sideways… it is not public*’, *(P24).*

Participants agreed on the universal benefit of more open discussion and knowledge about palliative care in providing a ‘*brace*’ (P21) and a ‘*mission plan*’ (P15) preventing being ‘*blind sighted*’ (P22) by life events and making decisions at particularly stressful times.

*‘No one person is going to get through life, without having to have those conversations’. (P11)*

However, recognition of the societal barriers to holding open conversations were identified. Fear of saying the wrong thing, of highlighting what people are no longer able to do and being responsible for exacerbating an already difficult situation were deemed obstacles to openly discussing palliative care. The prohibitive nature of these discussions was outlined by participants across faculties. A participant voiced his fear and perceived lack of skill;

*‘Because they might get quite upset and how to comfort them? Because there’s not an awful lot you can say if you know that person is going to die’. (P17))*

Throughout the interviews, participants expressed a lack of confidence in holding conversations relating to palliative care and the context of that care. However, for a participant who as a carer lived with illness and disability, different norms were acknowledged where discussions were uninhibited and began at an early age. For this family, open discussion was considered protective in empowering the young person and lessening the burden for the person who was ill;

*‘there never was a point where they (relative) could wait and talk about something or like maybe I’ll leave that for another year because you don’t know what’s going to happen’. (P11).*

Outside the context of the home, fear of being ‘*morbid*’ (P07) or ‘*fatalistic’* (P11) prohibited open discussion. A feeling that in some way by talking about palliative care they could be wishing illness on themselves or others was reported by several participants.

However, the perceived benefits of a community that is more knowledgeable of palliative care were considered multifaceted in allowing people to identify roles, to signpost to services and to provide an environment in which care is maintained. More openness might remove “*the elephant from the room*” (P07), thus preventing more social isolation of the person with life limiting illness and the family. In keeping with the focus of her discipline, a student in Speech and Language Therapy identified empowering communities to effectively communicate with the dying as a core goal of palliative care;

‘I *think it’s really knowing how to support people socially… like the difference that it can make for someone to be dying and have a great social circle, vs. dying and having virtually no social support, is quite traumatically different’. (P08).*

A change in culture that allowed more open discussion was considered the optimum context for public involvement with palliative care;

‘*Trying to come to a point where you can have safe debates and safe discussions without offending and maybe making judgements’. (P20).*

The role of the media as a trigger and means of disseminating information was supported by all participants, ensuring that information could be ‘*consumed in an instant*’ (P23) short and to the point, whether by leaflet, YouTube video, photography or television advertisements. Social media platforms such as Twitter, Facebook, WhatsApp and Snap Chat were all suggested as possible mediums. Participants felt that this was where young people spent a lot of their time and where they picked up information about a wide variety of issues. Social media could be used to garner interest and follow up with specific events;

*‘You could send an invite out on Facebook and that would reach everybody…. I think people around my age, we’re never off the internet!’. (P21)*

Putting ‘care’ across was highlighted as a difficulty because it needed a gentle, softer introduction that was not normally associated with widespread campaigns. Another difficulty was that palliative care was not a specific disease or behaviour but was a part of all diseases, a suggestion was made that it be included as an adjunct to education on all life-threatening illness,;

*‘Because it’s not an illness, so say, for example, depression is one type of illness, but palliative care incorporates loads of illnesses, so it would be hard to explain what kind of illnesses are in the palliative care range’. (P02).*

The general belief was that the public health input should be more about getting conversations started, planting the seed that would encourage them to look for information voluntarily.

### Organisational and policy influences

Organisations such as school, university and church were seen to have a potential role in promoting more positive attitudes to palliative care through normalising palliative care and providing connections for future engagement. Participants recommended the need to have a grass roots approach by bringing death and dying into the formal school system which was believed could in some way provide a ‘*brace*’ (P21*)* for later life. This was rationalised at three levels, first, the subject of end of life could be incorporated into the school where young children had the opportunity to process information especially related to sensitive subjects;

*‘The conversation happens with the teacher, but they can go into the playground or even just within the school classroom…and it just generates more creativity for them to understand how certain things work in life’. (P05).*

Secondly, the education system provided support structures and a safe place within which to discuss such topics. A gentle introduction was advocated and should involve making education more reflective of universal issues such as death and end of life care;

*‘In school you don’t really learn about death and I think sometimes you learn a lot of things that are irrelevant’. (P08)*

And finally, while young people at university were seen to have busy lives, they also had attributes such as ‘*youthfulness and energy*’ (P18) ‘*comfort and insight*’ (P19). The university was seen as a possible setting for disseminating knowledge relating to palliative through the distribution of leaflets in common areas and the use of performance art to aid understanding and make palliative care relatable.

Faith and church were seen by some participants as resources that allowed for easier engagement with the subject and practice of palliative care by creating a platform upon which discussions could be introduced and connections created. Religion was perceived as having provided a degree of hope in a very tense and uncertain time;

*‘The faith structure was a way to not make it feel it was final, or it was a scary thing. But I know not everybody has that sort of relationship with religion’. (P11)*

Governmental policy relating to the centralisation of health services further strengthened the need for communities to understand how to access palliative care services for themselves or family members;

‘*When you look at the way that hospitals and facilities are closing and are no longer available to people in certain areas, you kind of need to understand what facilities are there and what aren’t there and if something happens to your family, or a family member or a friend, that you’re not going in completely green and not finding everything out in one go, when you’re in a really stressful, emotional situation’. (P11).*

Wide and consistent dissemination of information involving whole communities was suggested by a participant with some realisation that, while the immediate benefits may not be immediately apparent, they would emerge at a population level.

‘*Realistically everyone will not get on board, that’s the truth. But a number of people will get on board and you might not see the result in five years, but if the community is consistent, they will see the result – it could take ten years and they could keep improving on it’. (P01).*

Participants identified influences on understanding of palliative care and potential targets for interventions at multiple societal levels. The embedded nature of influences was apparent in how participants understood palliative care, with individual engagement at the very centre determined by cultural influences at the outermost layer of the SEM (Fig. 1). In the absence of individual knowledge, cultural norms provided a safety net and a guide as to who, when and how they should engage with palliative care.

## Discussion

This study is the first study to explore in-depth how young adults perceive palliative care and identify what factors affect their understanding and engagement. Influenced by a prevalent culture that placed palliative care out of their reach and relevance unless directly affected, young adults in this study had a limited understanding of palliative care. They related what they had seen, heard and experienced in their families, community and on study placement which revealed a much narrower scope of palliative care than advocated in global policy and academia. The family as the context of experiences was significant in participant accounts, dictating the level of understanding and potential for engagement. At the intrapersonal and interpersonal levels the study findings have the potential to inform the practice of palliative care, highlighting how experiences, especially within families, impact young adults’ understanding of palliative care. Discussions with young people should therefore bear in mind that age may be a lesser determinant of comprehension and engagement individual experience.

Also highlighted were the fears, misperceptions and confusion as to what exactly palliative care entails, this has been identified in studies with patients, [35–37] carers, [38] health professionals [39, 40] and the general public [12, 41]. The responsibility for achieving clarity lies with palliative care professionals who should be tasked with constructing a simple and unequivocal explanation of palliative care and disseminate this message in a systematic way through all societal layers using available public health systems.

The term palliative care itself was not an issue for participants, rather the context of care and association with death was perceived as a source of societal discomfort. This association mirrors findings in studies among lay populations [12, 42], and palliative care researchers and academics [15]. Contrary to the WHO definition of palliative care [43] that emphasises palliative care as active care, participants’ perceptions were that it should be introduced only when every other option had failed, implying that it was something to which people succumbed rather than actively chose. Approaching caregiving from a managerial model with a sense of control gained from getting the job done has been identified among male carers [44]. This may explain the wish for information on how to ‘do’ relationships and ‘manage situations ‘among some of the male participants. A study of caring at the end of life found almost equal numbers of males and females in the young cohorts of carers [45]. If the misperception regarding palliative care is that nothing can be done, young male adults may be reluctant to avail of services for themselves or as surrogates. There is a need for investigation of gender needs and strategies to enhance engagement with palliative care, not just specifically in health education but on a more societal level.

Participants suggested that individually they were comfortable talking about palliative care and specifically death, dying and grief as it was, for most participants, an abstract and unexplored issue. The idea that ‘I personally have no problem talking about it’ but everyone else has, is a finding in studies undertaken with the public, [46–49] researchers and academics [15]. If all individuals are happy to talk about issues relating to palliative care, it is difficult to see why as an aggregate group society is not. Results from this study framed by the Socio- Ecological Model [28] shed light on this dichotomy. Palliative care was perceived as stages of individual private loss; loss of dependency, loss of goals, and loss of relationships and finally loss of persons. Perceived as a private experience and a fragile situation, more open discussion could be seen as potentially causing further harm. The embedded nature of influences suggests that sharing of knowledge by the palliative care sector at the organisational level could impact community confidence in discussing severe illness, death and loss. Assets can then emerge that could help extend the reach of palliative care. This can be achieved without minimising the unique experience of the individual at the centre of care.

The internet and particularly social media were the ‘go to’ for information, while older more experienced family and friends demonstrated the behaviour to follow. Recent empirical evidence in Europe and the USA demonstrated that information on internet sites is unstandardised and can be based on particular agendas with important omissions relating to core aspects of palliative care [50–52]. Analysis of You Tube videos relating to palliative care identified the potential for misinformation to be disseminated [53]. Yet findings from this study suggest the use of established platforms such as the education system and social media can provide not only the means but the impetus for engaging with palliative care. Similar requests for interactive discussion using social media were reported in a study of young adults’ perceptions of infection prevention of sexually transmitted disease and Human Immunodeficiency Virus, where participants felt that sensitive subjects could be tackled on an interactive forum such as social media [54]. Informing a public health approach, this study has identified a platform for disseminating information but with it a responsibility for ensuring that information accessed online is consistent with the philosophy of palliative care and accurately reflects its goals.

Intervention studies aiming to educate adolescents regarding palliative care undertaken in schools [55] and church settings [56] have demonstrated positive results in increasing knowledge about the benefits of palliative care and influencing attitudes towards people with life limiting illness. In this study participants identified the university as a place for disseminating information on palliative care and influencing attitudes, making resources already in place more responsive to actual issues facing communities. Providing the opportunity and space for reflection was deemed important by participants in this study, a finding also reported in a community based initiative that aimed to bring conversations about living and dying into the public domain [57]. Participants in this study perceived themselves as open to learning more about palliative care with the interview giving them an opportunity to reflect on their attitudes. Reflecting on attitudes is a central part of the ‘Last Aid’ course, a European initiative that mirrors the goals of a public health approach to palliative care by influencing individual capabilities to provide a broader more positive community response to socially support people at the end of life [58, 59]. The perceived potential of educational initiatives to influence attitudes and enable community participation in palliative care was evident in this study, with participants suggesting that the education system would provide an avenue within which palliative care could be taught, spoken about and placed within a safe environment. Allowing young adults a say in determining and communicating policy that introduces palliative care into the education system can make its introduction seamless and responsive. This might lead to a normalisation of palliative care with tangible improvements in understanding.

### Limitations and strengths

This study was undertaken in one university with a limited sample in terms of ethnicity and sociodemographic background. It gives a snap shot of views of an educated cohort of the young public in one region. Young adults outside the university setting from other ethnic, socioeconomic and educational backgrounds need to have their voices heard as they may have differing perceptions of palliative care and could point to other determinants that need to be addressed to allow for their engagement.

The study provides a previously unexplored insight into young adults’ understanding of palliative care. The use of the Socio-Ecological model as a theoretical underpinning allowed for consideration of a breadth of influences, not only on present understanding but on how public health and palliative care can, in partnership, promote better understanding and make palliative care relevant and relatable to young adults.

## Conclusion

In this study palliative care was viewed as a private issue, the fragility of the situation in which palliative care was perceived to take place meant that engagement could be seen as intrusive for those not directly involved. Public discussion had the potential of bringing a commonality to something that was inherently irreversible, devastating and private. Despite this, young adults demonstrated a readiness to engage with palliative care but felt inhibited by a culture that placed restrictions on whom, how and when engagement should take place. The study highlights how the SEM could provide a framework for a public health approach to understanding and engaging with palliative care, guiding interventions aiming to change these perceptions. The dynamic and bidirectional influence of the various societal levels provides opportunities for both palliative care and public health to influence public understanding and prompt societal action required to meet the unprecedented need for palliative care.

## Supplementary Information

**Additional file 1.** Interview Items.

## Data Availability

The data sets generated and analysed during the present study are not publicly available owing to consideration of the interviewees' privacy but are available from the corresponding author on reasonable request and form.

## Abbreviations

SEM: Socio-Ecological Model
