# Supplementary material for: Young adults understanding and readiness to engage with palliative care: extending the reach of palliative care through a public health approach: a qualitative study
Source: BMC Palliat Care. 2021 Jul 28;20:120. doi: 10.1186/s12904-021-00808-0 (PMC8320215; doi:10.1186/s12904-021-00808-0)
Supplement: Supplementary file 1 — Additional file 1. Interview Items. [file 12904_2021_808_MOESM1_ESM.docx]

*Young adults understanding and readiness to engage with palliative care: Extending the reach of palliative care through a public health approach: A qualitative study*

*Anita Mallon^1^ PhD. Postdoctoral Researcher.

^1^Institute of Nursing and Health Research, Ulster University, Shore Road Newtownabbey. Belfast, Co. Antrim. BT37 0QB, Email: [mallon-a16@ulster.ac.uk](about:blank). ORCID 0000-0002-2039-4102

Felicity Hasson^1^ PhD Senior Lecturer,

^1^Institute of Nursing and Health Research, Ulster University, Shore Road Newtownabbey, BT37 0QB, Northern Ireland. Email: [f.hasson@ulster.ac.uk](about:blank) ORCID: 0000-0002-8200-9732

Karen Casson^1^ PhD Lecturer in Health Promotion and Public Health

^1^Institute of Nursing and Health Research, Ulster University, Shore Road Newtownabbey. Belfast, Co. Antrim. BT37 0QB, Email: [k.casson@ulster.ac.uk](about:blank) , ORCID 0000-0002-6178-4748

Paul Slater^1^ PhD Lecturer/ Statistician

^1^Institute of Nursing and Health Research, Ulster University, Shore Road Newtownabbey. Belfast, Co. Antrim. BT37 0QB, Email: pf.slater@ulster.ac.uk ORCID: 0000-0003-2318-0705

Sonja McIlfatrick^1^ PhD Professor of Nursing/ Head of School of Nursing

^1^Institute of Nursing and Health Research, School of Nursing, Ulster University, Shore Road Newtownabbey, BT37 0QB, Northern Ireland. Email [sj.mcilfatrick@ulster.ac.uk](about:blank) ORCID 0000-0002-1010-4300

*Corresponding author

Additional File (1) Interview Items

1. Before filling in the survey had you heard of palliative care? What do you understand by palliative care?
2. How did you come to know about palliative care?
3. Given that you understand palliative care to be….how do you feel about talking about palliative care?
4. Is there a stage in life when the subject of palliative care should be introduced, what barriers might be encountered?
5. So your perception of palliative care is that it is about….. Tell me what you would like to know about palliative care?
6. The survey showed that the majority of young students want more information on palliative care. What way can this information reach young people?
7. Over the years many subjects have been tackled via public campaigns, can you think of any that you felt tried to reach out to you as a young person? What made them successful? Transfer to palliative care?
8. How might your community benefit from knowing more about palliative care?
